# Comparative Analysis of Transcriptomes from Secondary Reproductives of Three *Reticulitermes* Termite Species

**DOI:** 10.1371/journal.pone.0145596

**Published:** 2015-12-23

**Authors:** Franck Dedeine, Lucy A. Weinert, Diane Bigot, Thibaut Josse, Marion Ballenghien, Vincent Cahais, Nicolas Galtier, Philippe Gayral

**Affiliations:** 1 Institut de Recherche sur la Biologie de l’Insecte, UMR 7261, CNRS—Université François Rabelais, 37200, Tours, France; 2 Institut des Sciences de l’Evolution, UMR 5554, Université de Montpellier—CNRS—IRD—EPHE, Montpellier, France; Institute of Plant Physiology and Ecology, CHINA

## Abstract

Termites are eusocial insects related to cockroaches that feed on lignocellulose. These insects are key species in ecosystems since they recycle a large amount of nutrients but also are pests, exerting major economic impacts. Knowledge on the molecular pathways underlying reproduction, caste differentiation or lignocellulose digestion would largely benefit from additional transcriptomic data. This study focused on transcriptomes of secondary reproductive females (nymphoid neotenics). Thirteen transcriptomes were used: 10 of *Reticulitermes flavipes* and *R*. *grassei* sequenced from a previous study, and two transcriptomes of *R*. *lucifugus* sequenced for the present study. After transcriptome assembly and read mapping, we examined interspecific variations of genes expressed by termites or gut microorganisms. A total of 18,323 orthologous gene clusters were detected. Functional annotation and taxonomic assignment were performed on a total of 41,287 predicted contigs in the three termite species. Between the termite species studied, functional categories of genes were comparable. Gene ontology (GO) terms analysis allowed the discovery of 9 cellulases and a total of 79 contigs potentially involved in 11 enzymatic activities used in wood metabolism. Altogether, results of this study illustrate the strong potential for the use of comparative interspecific transcriptomes, representing a complete resource for future studies including differentially expressed genes between castes or SNP analysis for population genetics.

## Introduction

Termites (Blattodea, Termitoidae) constitute an ecologically and evolutionary diversified group of social insects (>2600 species) that share a common ancestry with cockroaches [1]. The ecological success of termites is often attributed to the combination of their sophisticated social organization with their unique ability to feed on recalcitrant plant matters such as wood (lignocellulose) [2]. Lignocellulose digestion relies on a complex enzymatic system which is synthesized by termites and a diverse intestinal microbial community composed of numerous prokaryotes and, in some termites, unicellular eukaryotes (flagellated protists) [3]. Termites are major decomposers in many tropical and subtropical ecosystems and therefore, are crucial for recycling organic matter [2]. Conversely, some termites are pests, causing serious damage to human-built structures and woody plant crops.

Aside from a specialized nutritional regime, another characteristic of termites allowing them to successfully diversify worldwide is their sophisticated social organization. As in other social insects, such as social Hymenoptera (ants, some bees and wasps), termites live in complex societies where individuals are morphologically, physiologically and behaviorally specialized into distinct castes. The castes work together to accomplish specific and complementary tasks within a colony. Division of labor among castes is the key to efficient colony development, survival and reproduction. The social organization of termites also represents a primary reason why termite infestations can be difficult to control and eradicate. Therefore, a detailed understanding of the expressed genes of termites is not only interesting for academic research but is also essential in the development of new termite-specific insecticides [4].

The acquisition of genetic data in termites and their gut microbial community has been of recent interest to the scientific community. This is mostly due to the development and accessibility of new sequencing technologies such as 454 pyrosequencing and Illumina sequencing [4]. To date, (meta-) genomic and (meta-) transcriptomic studies in termites have been principally aimed at identifying host and/or symbiont genes underlying lignocellulose digestion [5–12], caste differentiation [13–17], reproduction [18] or defense [19]. Large-scale EST libraries have also been constructed in a few distantly related termite species belonging to different families for comparative purpose [13]. Despite these efforts and the recent publication of the first two termite genomes [20,21], genetic data are only available for a limited number of termite species and comparative studies remain scarce. The diversity and identity of genes expressed have rarely been compared between closely related species.

*Reticulitermes* (Rhinotermitidae) represent an important genus of termites with multiple pest species, particularly in temperate regions [22]. They have cryptic nesting habits and form complex colonies with diffuse nests and multiple feeding sites connected by underground tunnels [23]. Termite colonies are typically founded by a single pair of winged reproductives (i.e., the “queen” and the “king”) following a nuptial flight. However, reproduction is not always reserved only to the primary couple within colonies. Another type of reproductives can indeed differentiate among the offspring of the primary couple. Such secondary reproductives are called ‘nymphoid neotenics’ when they differentiate from nymphs and ‘ergatoid neotenics’ when they differentiate from workers. Although these two types of neotenics are morphologically different, both are wingless and have no pigmentation; they stay in their native colonies where they may replace or supplement the primary couple’s reproduction. The presence of productive neotenics within colonies has tremendous genetic and dynamic impacts on colonies [23], and some authors have argued that the acquisition of this caste has played a major role in the evolution of social life [24,25]. In *Reticulitermes* termites, the number of neotenics is extremely variable among and within species, and several studies have argued that a high number of neotenics could improve the capacity of colonies to develop and disperse in urban areas [23,26,27]. Despite their importance, the conditions under which neotenic reproductives differentiate within colonies as well as the molecular mechanisms underlying such a differentiation remain unknown. In comparison with other castes (workers, soldiers, primary reproductives), only a few studies focus on determining the genes expressed in neotenic reproductives of termites [15,28,29].

The present study compares the transcriptome content obtained from 13 nymphoid neotenic females of three *Reticulitermes* species: two West European species, *R*. *grassei* and *R*. *lucifugus* [30], and one North American species, *R*. *flavipes*, that has been introduced to France [26]. Eleven out of the thirteen transcriptomes analyzed in this study (i.e., those obtained in *R*. *flavipes* and *R*. *grassei*) were obtained in a previous study [31]. This dataset was used for SNP detection and population genomics inference only [32,33] and no gene content and functional analysis was performed. Using the same protocol, two additional transcriptomes were generated from nymphoid female neotenics collected in two distinct colonies of *R*. *lucifugus*. BLAST analysis allowed functional and taxonomic assignation of the predicted contigs, assembled from the whole species from pools of individuals and compared between termite species. Functional analysis of contigs associated with wood degradation was performed using Gene Ontology (GO) term analysis and contributed to the characterization of *Reticulitermes* transcriptomes.

## Materials and Methods

### Termite samples

We sampled 13 termite colonies representative of 3 *Reticulitermes* species: 9 colonies of *R*. *grassei*, 2 colonies of *R*. *flavipes* and 2 colonies of *R*. *lucifugus*. All samples were collected in 2010 in France from 11 locations (Table 1). None of these termite species are endangered or protected, and no specific permission was required for collecting them since they were taken from unprotected areas. Five hundred to 1000 individuals per colony were brought to the laboratory where they were maintained for 90 days under controlled conditions in their original wood piece at 25°C and 80% humidity. For each colony, the transcriptome of a single fecund nymphoid neotenic female was obtained using the protocol described below. The transcriptomes obtained from *R*. *grassei* and *R*. *flavipes* females were obtained and assembled in a previous study [31]. In this study, we used a single nymphoid neotenic female isolated from 2 different colonies of *R*. *lucifugus* for further RNA isolation, cDNA library construction and transcriptome sequencing (see below).

**Table 1:** Information and sequencing results of nymphoid neotenic female termites used in this study.

Identification name	*Reticulitermes* sp.	Locality	Latitude	Longitude	No of illumina reads (x10^6^)
GA15A	*R*. *grassei*	Pissos	44.307069	-0.778946	14.95
GA15B	*R*. *grassei*	Poitiers	46.580224	0.340375	44.77
GA15C	*R*. *grassei*	Gatseau	45.826772	-1.208771	27.21
GA15D	*R*. *grassei*	Gatseau	45.826772	-1.208771	37.83
GA15E	*R*. *grassei*	Ustaritz	43.395936	-1.45459	22.20
GA15F	*R*. *grassei*	Eguilles	43.5686589	5.354234	28.57
GA15G	*R*. *grassei*	Eguilles	43.5686589	5.354234	27.60
GA15H	*R*. *grassei*	Cugnaux	43.537141	1.344995	24.52
GA15I	*R*. *grassei*	Petit Bôo	44.353499	-1.042781	23.30
GA15K	*R*. *flavipes*	Olonne	46.536402	-1.772826	58.86
GA15L	*R*. *flavipes*	Oléron	45.9159335	-1.2716743	46.98
GA15M	*R*. *lucifugus*	Allauch	43.336148	5.4825739	43.65
GA15N	*R*. *lucifugus*	Le Rove	43.369912	5.252998	44.80

### RNA isolation

Total RNA was isolated independently from the whole body of a single neotenic using an adapted protocol using Guanidinium Thiocyanate-Phenol solution supplemented with glycogen [34]. Quality and quantity of total RNA were determined using agarose gel electrophoresis, NanoDrop spectrophotometry and analysis on Agilent bioanalyzer 2100 system using the Eukaryote Total RNA Nano assay. RNA isolation for the two other species *R*. *grassei* and *R*. *flavipes* used in this study was obtained with the same protocol [31].

### Transcriptome sequencing

For each sample, 5 μg of total RNA of *R*. *lucifugus* were used to build 3’-primed, non-normalized cDNA libraries. Although prokaryotic RNA might be present in the libraries since the gut was not removed before extraction, these transcripts were not specifically targeted. Oligo(dT)-primed first-strand synthesis and cap-primed second-strand synthesis were performed using the SMART cDNA library construction kit (Clontech, Mountain View, CA, USA). Libraries were sequenced using Genome Analyzer II (Illumina) with 5 tagged libraries pooled per lane. Fifty bp single-end reads were produced. The cDNA library construction and sequencing were performed by GATC biotech company (Constanz, Germany). After tag-removing, low quality bases, adaptors and primers were removed with SeqClean software (http://compbio.dfci.harvard.edu/tgi/) with default parameters. Reads used for this study originated from a previous work [31] on *R*. *flavipes* (Sequence Read Archive accession no. SRX565295 and SRX565296) and *R*. *grassei* (SRA accession no. SRX565297 to SRX565305), and from the present study for *R*. *lucifugus* (SRA accession no. SRX565306 and SRX565307).

### Transcriptome assembly

Transcriptomes were assembled by pooling the reads obtained from individuals belonging to the same species (N = 9 for *R*. *grassei* and N = 2 for both *R*. *flavipes* and *R*. *lucifugus*). Assemblies were performed using ABYSS [35] with Kmer set at 40, followed by two consecutive runs of CAP3 [36] as described in [37]. Contigs shorter than 100 bases were discarded.

### ORF detection

Complete and 5’- or 3’-truncated open reading frames (ORF) were detected using Prodigal software for metagenomic data [38] using standard genetic code. ORF with a stretch of N (undetermined nucleotides) inside the sequence were not discarded. When several ORF were detected on the same contig, only the longest was kept since it would more likely correspond to a true protein. The software Cd-hit [39] was used to remove ORF redundancy from our dataset by detecting sequences showing 100% identity in homologous regions.

### Orthology prediction

BLAST-based pairwise orthologs relationships between *R*. *grassei*-*R*. *flavipes*, *R*. *grassei-R*. *lucifugus*, and *R*. *flavipes-R*. *lucifugus* species pairs were first assessed by InParanoid 4.1 program using default parameters [40]. Non-redundant translated contigs of the 3 species displaying length > 100 bases were used for that purpose. MultiParanoid [41] was then used to analyze the orthology relationship of gene clusters between the 3 species.

### Taxonomic assignation

BLAST results from the annotation step were parsed to retrieve sequence identifier (GI) and NCBI taxonomic identifier (TaxID) from the NCBI database (ftp://ftp.ncbi.nih.gov/pub/taxonomy). Each contig was assigned to 5 taxonomic ranks (i.e., superkingdom, kingdom, phylum, genus and species). Contigs were assigned to Bacteria when the indicated superkingdom was ‘Bacteria’; they were assigned to Termites when the superkingdom was ‘Eukaryota’ and the kingdom was ‘Metazoa’; they finally were assigned to protists when the superkingdom was ‘Eukaryota’ and when the kingdom was not ‘Fungi, Viridiplantae and/or metazoa’. For protists, downstream taxonomic ranks (phylum, genus and species) were inspected manually to verify that each of them corresponded to a protist taxon.

### Functional annotation

Amino acid homologies of the non-redundant predicted ORFs were analyzed using BLASTp program [42] against the Genbank nr database (March 1, 2011). The first BLAST hits were kept and the minimum E-value was set at 0.001. HSP length cut off was 33 and the lower capacity filer was enabled. GI identifiers from BLAST results were used to retrieve UniProt IDs from the PIR Protein Sequence Database, the latter served to associate GO terms with our predicted ORFs. In addition, known protein signatures were detected using the software InterProScan [43] based on InterPro collection databases. Annotation steps were performed with Blast2go software V.2.2.6 [44] using default parameters. GO terms analysis was performed on the three termite species independently. Based on the KEGG metabolism pathways database V.64.0 [45,46] implemented in Blast2go, GO terms of biological processes were used to retrieve contigs belonging to the starch and sucrose metabolism process (map00500). GO results were displayed at GO level 2. A GO level referred to the hierarchical structure of the Gene Ontology as the number of GO terms between a given term and the Root Term of the ontology. Contigs assigned to a function related to the starch and sucrose metabolism process were further analyzed for the presence of hallmarks of Carbohydrate-Active Enzymes (CAZ) domains using CAZymes Analysis Toolkit (CAT) [47] with the CAZy database updated on 09/20/2013 [48]. In this software, the assignment method is based on both similarity search on proteins sequences and the presence of Pfam conserved domains (option ‘Pfam rules based annotation’).

### Quantification of transcript abundance

For each species, a pool of individual reads were mapped to the species transcriptome using BWA [49] with default parameters and served for FPKM (i.e., Fragment Per Kilobase per Millions fragments mapped) calculation using Cufflinks [50]. To avoid biases due to differences in read numbers across individual data sets, a random subsampling of reads was performed before pooling. A total of 14.95, 49.98 and 43.65 million reads were subsampled for each individual of *R*. *grassei*, *R*. *flavipes* and *R*. *lucifugus*, respectively. This number corresponded to the number of reads of the individual displaying the lower number of reads for the species. Only the 250 most abundant contigs per species (with higher FPKM values) with a Blast2GO hit were kept. As previously described, the functional annotation of these contigs was performed using Blast2GO. Preliminary analyses indicated that GO term levels 2 and 3 were not appropriated due to a limited number of contigs producing too few functional categories. The final analysis was therefore conducted at the GO term level 4.

## Results and Discussion

### Sequencing, assembly and annotation of transcriptomes

The numbers of illumina reads obtained for all of the 11 termite samples are indicated in Table 1. For each termite species, reads from conspecific individuals were pooled together before assembly (N = 9 for *R*. *grassei*, N = 2 for *R*. *flavipes* and *R*. *lucifugus*). The obtained species-level assemblies were comparable among species and exhibited high N50 values (Table 2). In total, 64,328, 65,814 and 79,404 non-redundant ORF were predicted in *R*. *flavipes*, *R*. *lucifugus* and *R*. *grassei*, respectively. As expected with non-model organisms lacking complete and well-annotated genome sequences, only a third of ORF showed a significant BLAST hit in the nr protein database. After the functional annotation performed with Blast2Go program, only a small fraction (6–8%) of the initial ORF set could be assigned with one or more GO terms (Table 2). Whatever the number of individual reads sets pooled together (2 or 9), the number and quality of the obtained contigs were similar among species, suggesting that pooling reads from 2 individuals produced satisfactory results.

**Table 2:** Assemblies of transcriptomes, ORF predictions and functional annotations for interspecific comparisons and Gene Ontology analyses. Contigs < 100 bases were removed from analyses.

Species	*R. flavipes*	*R. grassei*	*R. lucifugus*
No of contigs	93,420	111,549	91,280
N50	998	1,041	1,067
No. of predicted ORF	64,342	79,640	65,855
Non-redundant ORF	64,328	79,404	65,814
No of ORF with BLASTp hit	19,375 (30.1%)	21,671 (27.3%)	20,377 (31%)
No of annotated ORF (GO term)	5,389 (8.4%)	4,922 (6.2%)	5,214 (7.9%)

### Orthology relationships

**Fig 1:**
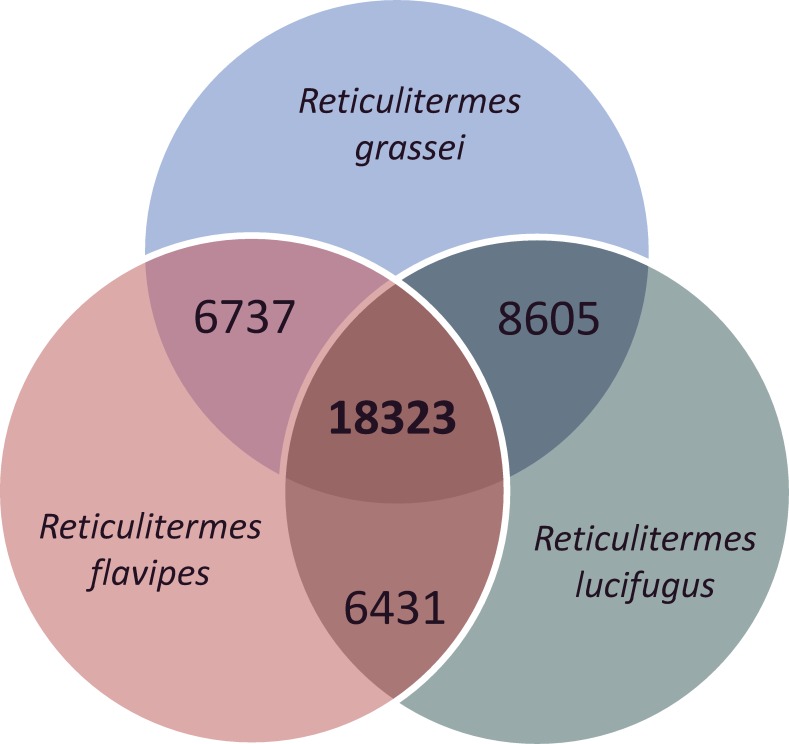
Orthology relationships between *R*. *grassei*, *R*. *flavipes* and *R*. *lucifugus* contigs. The number of orthologous gene clusters is indicated inside Venn diagram.

Gene clusters were identified within each species from transcript data. The presence of homologous clusters between species (i.e. orthologues) was assessed to better understand the genetic relationships between the 3 termite species. For each species, transcript clusters were composed of representative unigenes (i.e. alternative transcripts derived from a single locus) or transcripts derived from young duplicated genes (i.e. paralogues). Interspecific comparison showed that a large number (18,323) of orthologous genes clusters (i.e. homologous genes clusters identified in other species) were detected in the 3 species (Fig 1). Orthologous genes were more abundant between *R*. *grassei* and *R*. *lucifugus* (8,605) than between the two other pairs of species (6,737 between *R*. *grassei* and *R*. *flavipes*, and 6,431 between *R*. *lucifugus* and *R*. *grassei*). This result is in accordance with the phylogenetic relationships between these termite species [30]. The North American species, *R*. *flavipes*, is indeed distantly related to the two European species, *R*. *grassei* and *R*. *lucifugus*, which are closely related and probably sister species. Transcripts of gut microbiota may also reinforce this phylogenetic relationship between the taxa, albeit to a lesser extent since they do not contribute much in terms of contig numbers (see next paragraph). This hypothesis is supported by recent studies showing how microbial communities are usually more similar between closely related species than distant species [51–54].

### Taxonomic assignment

Table 3 shows the taxonomic distribution of the contigs displaying a significant BLAST hit of their coding sequence against the nr protein database. In total, 41,287 contigs were assigned. Most of them (94.9%) were assigned to the termite genome, whereas the remaining contigs were assigned to diverse lineages of microorganisms (i.e. protists, bacteria, archeae, virus) (3.9%), fungi (0.9%) or plants (0.4%). Contigs assigned to fungi and plants most likely represent environmental contaminations since no endosymbiotic association has been described so far between these organisms and *Reticulitermes* termites. Most contigs assigned to microorganisms were probably expressed by diverse microorganisms living in the hindgut of *Reticulitermes* termites. These well-known microbial communities are composed of protists (two main lineages: Parabasalia and Oxymonadida), Bacteria (the most abundant: Spirochaetes, Bacteroidetes, Firmicutes and Elusimicrobia), methanogenic Archeae (Methanobacteriaceae family) and bacteriophage virus infecting Spirochaetes [3,55,56].

**Table 3:** Taxonomic assignation of contigs obtained in the three termite species.

Species		*R. flavipes*	*R. grassei*	*R. lucifugus*	Total
No. of transcriptomes		2	9	2	13
No. of contigs (%)	All	13,889 (100.00)	14,453 (100.00)	12,945 (100.00)	41,287 (100)
	Termites	12,806 (92.11)	13,620 (94.05)	12,798 (94.05)	39,224 (94.87)
	Microorganisms ^a^	1,006 (7.23)	438 (3.02)	95 (0.73)	1,539 (3.92)
	Protists	893 (6.42)	300 (2.07)	39 (0.30)	1,232 (3.14)
	Bacteria	101 (0.72)	120 (0.82)	46 (0.35)	267 (0.68)
	Archeae	6 (0.04)	9 (0.62)	3 (0.02)	18 (0.04)
	Viruses	6 (0.04)	9 (0.62)	7 (0.05)	22 (0.05)
	Fungi	39 (0.28)	302 (2.08)	26 (0.20)	367 (0.93)
	Viridiplantae	38 (0.27)	93 (0.64)	26 (0.20)	157 (0.40)

^a^Contigs of microorganisms include those of Protists, Bacteria, Archeae and Viruses.

Several reasons could explain the low proportion of microbial contigs in our dataset. First, contigs assigned to prokaryotes were particularly scarce (< 1% for the 3 termite species), probably because the cDNA library protocol underwent an enrichment of mRNA based on the existence of poly-A tails, which are mostly absent in prokaryotic transcripts. Second, as expected with non-model organisms, which lack complete and well-annotated genome sequences, expressed genes in gut microbial communities of termites have not been fully characterized yet. Therefore, the genomic database is likely incomplete and could thus result in an underestimation of microbial genes. Third, all transcriptomes were generated from nymphoid neotenic females. Like other castes or developmental stages in subterranean termites, reproductive castes do not necessarily feed on the wood themselves and instead are fed by workers who provide them with nutrient-rich salivary trophallactic transfers [57]. Since the gut microbiota might be not essential for extracting nutrients from wood in these secondary reproductives, microorganisms could be less abundant in the hindgut of reproductives compared to that of wood-feeding castes. This hypothesis is supported by previous work in primary reproductives (alates) in 3 species of *Reticulitermes* [58], as well as even earlier work in *R*. *flavipes* [15] which suggests a reduced microbiota in neotenic reproductives.

### Description of gene ontologies of expressed genes

**Fig 2:**
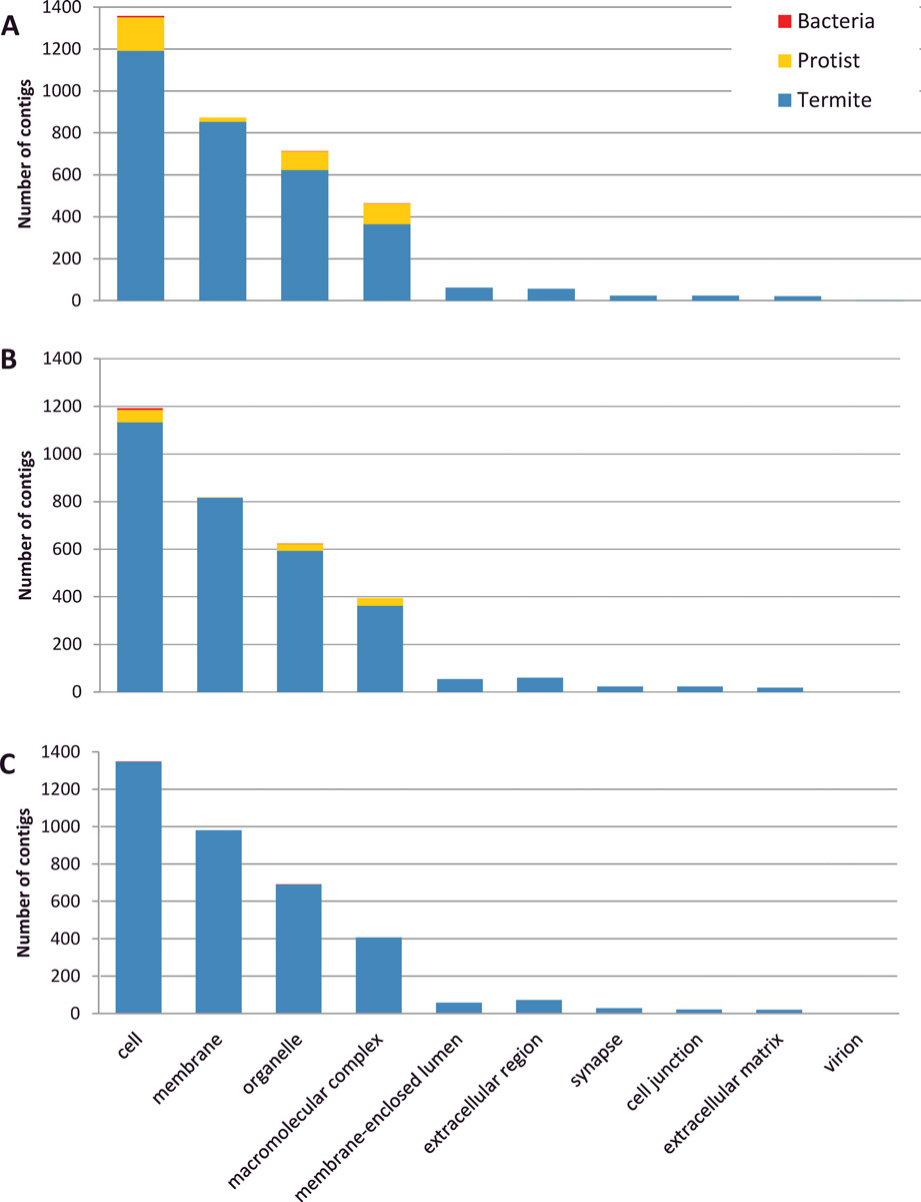
Gene Ontology analysis of Cellular Components (GO level 2). A: *R*. *flavipes*. B: *R*. *grassei*, C: *R*. *lucifugus*.

**Fig 3:**
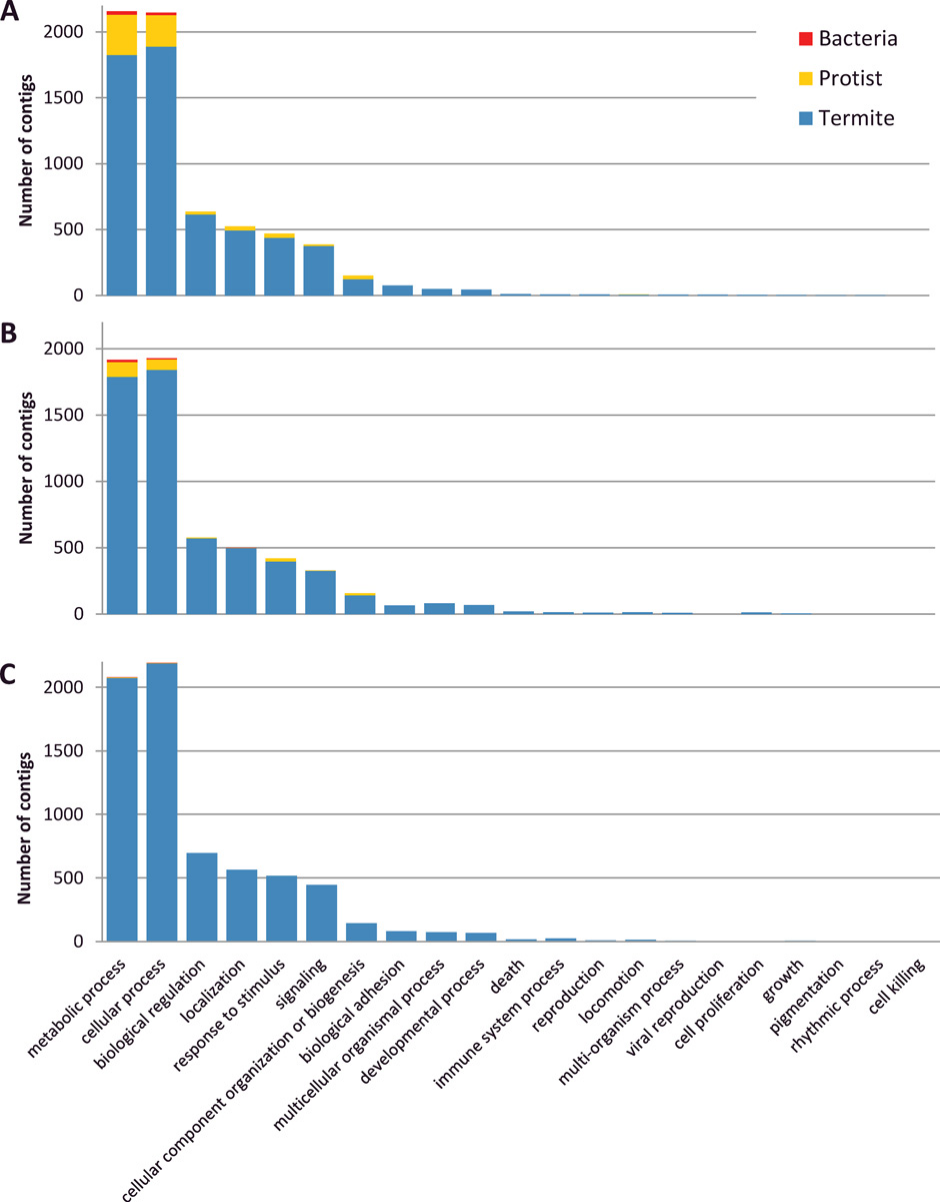
Gene Ontology analysis of Biological Processes (GO level 2). A: *R*. *flavipes*. B: *R*. *grassei*, C: *R*. *lucifugus*.

**Fig 4:**
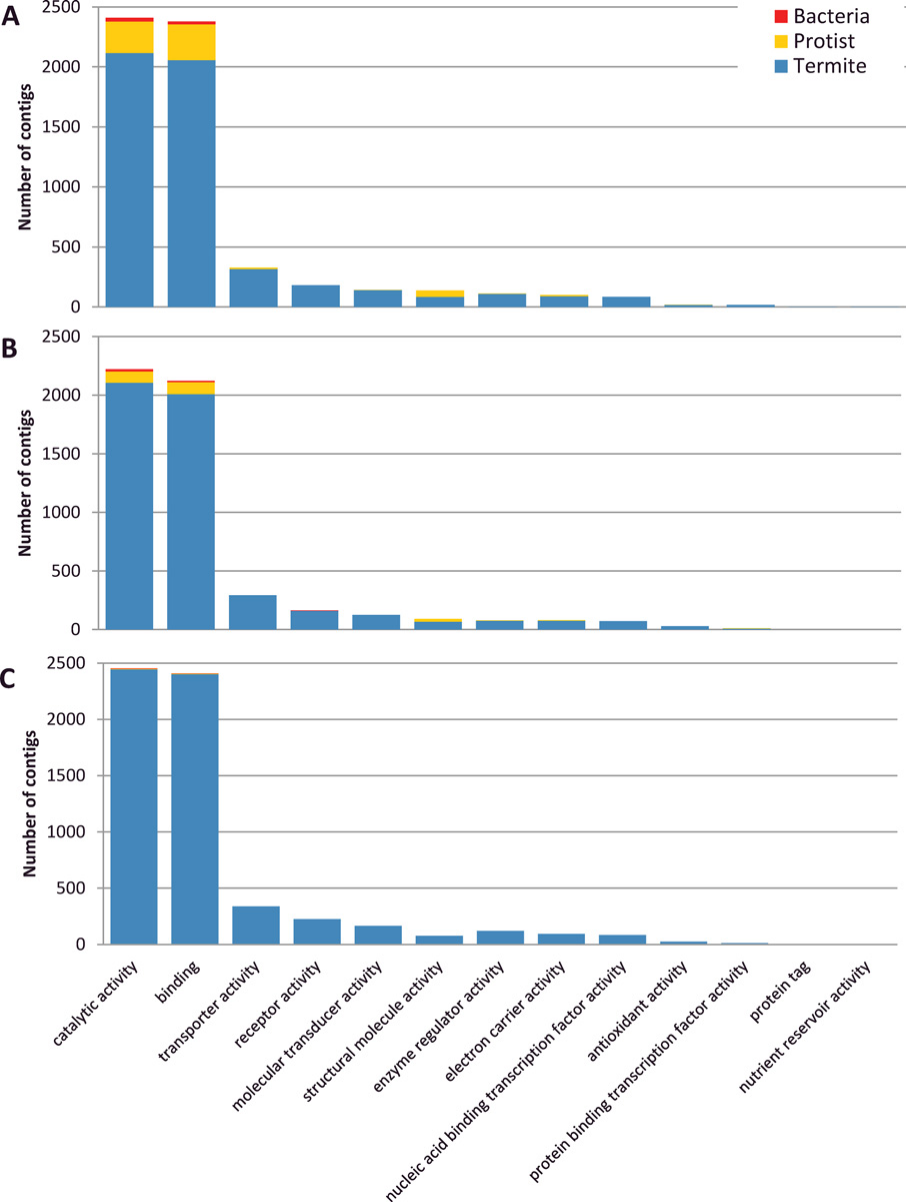
Gene Ontology analysis of Molecular Functions (GO level 2). A: *R*. *flavipes*. B: *R*. *grassei*, C: *R*. *lucifugus*.

The function of assembled contigs was evaluated by retrieving GO terms according to their termite, protist or bacterial origin. The 3 descriptive ontologies ‘cellular components’, ‘molecular function’ and ‘biological process’ were analyzed. For the cellular components ontology, 10 localizations were found. Transcripts products mainly localized in cell (37–38% depending on termite species), membrane (24–27%), organelle (19–20%) and macromolecular complex (11–13%) (Fig 2). Twenty-one classes of biological processes were found with 2 dominant classes corresponding to metabolic (30–32%) and cellular processes (31–32%) (Fig 3). Finally, 13 GO terms were found in ‘molecular function’, among which putative catalytic activity (40–42%) and binding (40%) were the most abundant (Fig 4). A very similar distribution of GO terms for the 3 types of ontologies was observed between the 3 termite species, suggesting unbiased transcriptome assembly. The relative taxonomic distribution (termite, protist, bacteria) was consistent among the 3 ontologies and with the total number of contigs (Table 3). GO analysis showed that protists accounted for a significant part of cellular components (mainly cell, organelle and macromolecular complex) of biological processes (metabolic and cellular process) and molecular functions (catalytic activity, binding and structural molecule activity).

**Fig 5:**
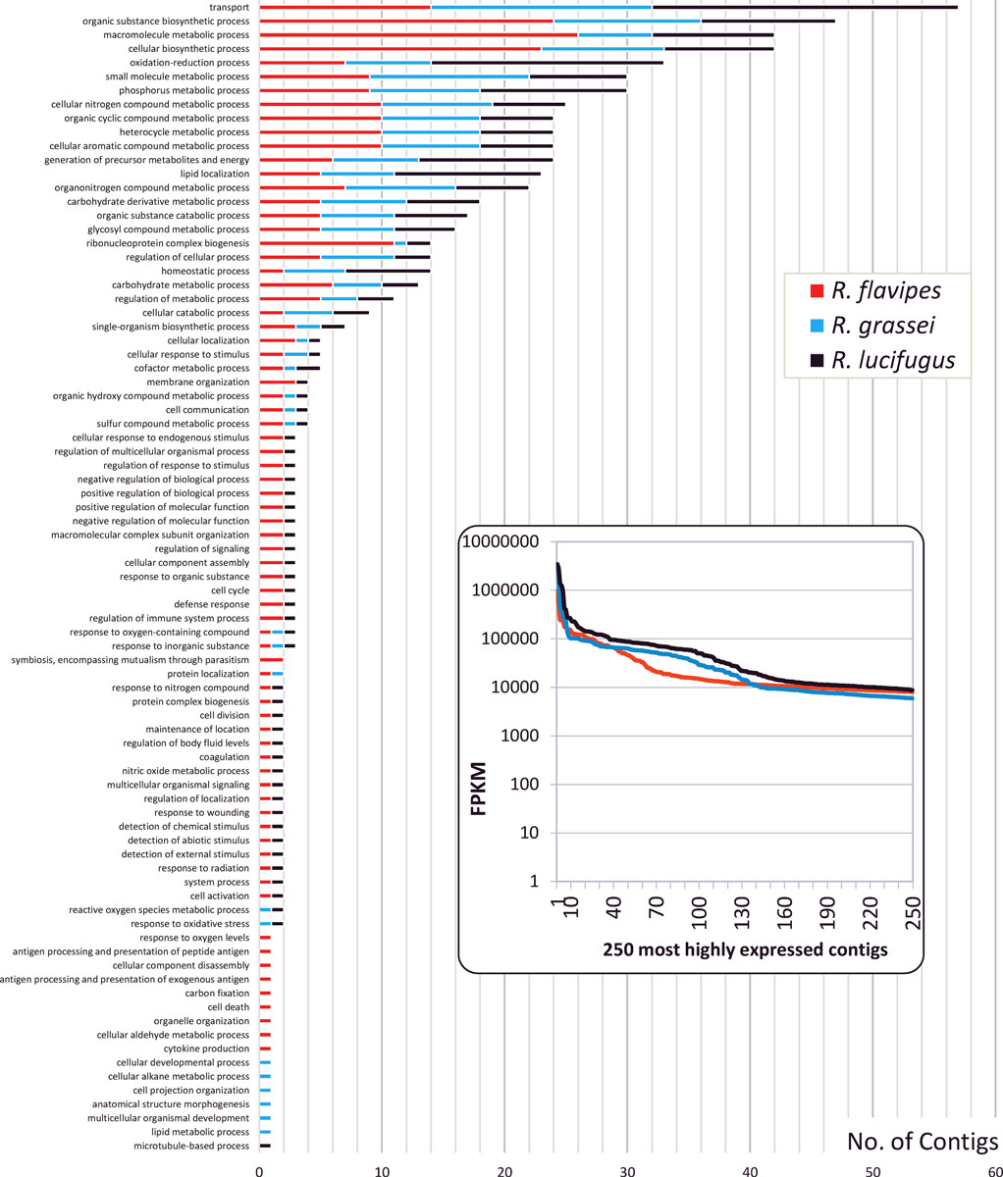
Distribution of the 250 most expressed contigs across their biological functions detected by GO term analysis at the GO level 4. The relative abundance of contigs estimated by FPKM is shown in the box.

The putative biological functions of the 250 most expressed contigs were studied for each of the 3 termite species (Fig 5). These 750 contigs corresponded to highly expressed transcripts, having a FPKM value ranging from 5,940 in *R*. *grassei* to 3,448,530 in *R*. *lucifugus*. Eighty-three functional categories were assigned in total among the 3 termite species. The 31 functional categories displaying the most numerous contigs were related to metabolic process whereas the 52 remaining categories corresponded to diverse other biological functions. This result appeared consistent with the global analysis of transcriptomes (Fig 4), and suggests that the most expressed genes have metabolism-related functions.

### Contigs putatively associated with wood-degradation enzymes

**Fig 6:**
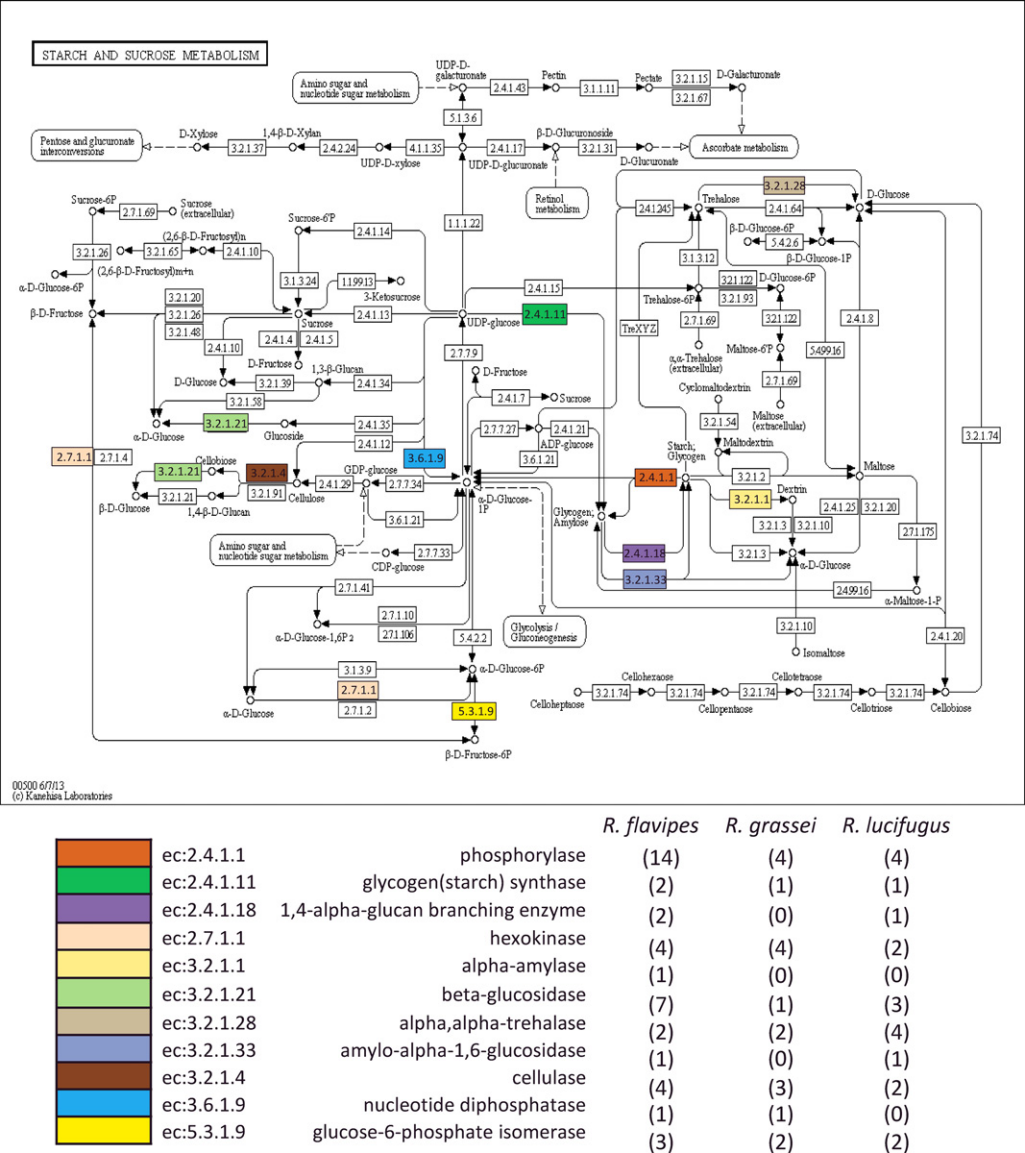
Contigs of *R*. *flavipes*, *R*. *grassei* and *R*. *lucifugus* displaying putative enzymatic activities involved in starch and sucrose metabolism. Top panel: termite contigs (colored boxes) mapped on the starch and sucrose metabolism KEGG map (black and white boxes). Bottom panel: number of contigs (brackets) associated to the 11 putative functions of starch and sucrose metabolism.

Transcriptome annotation was used to retrieve contigs associated with functions belonging to the starch and sucrose metabolism. Some of these may be involved in cellulose degradation [59]. The complete list of contigs displaying functions related to starch and sucrose metabolism and their amino acid sequences are shown in supplementary information (S1 Table and S1 Dataset). Fig 6 shows the enzymatic processes detected in our dataset and plots them on starch and sucrose metabolism map. A total of 11 functions putatively associated with known enzymatic activities were found in the assemblies. Among these functions, we found contigs putatively associated with cellulytic activities. Cellulase is a general term for cellulytic enzymes, of which three main classes are recognized on the basis of the mode of enzymatic actions and substrate specificities: endoglucanases (EGs; EC 3.2.1.4), cellobiohydrolases (CBHs; EC 3.2.1.91) and β-glucosidases (BGs; EC 3.2.1.21). These three categories of enzymes work synergistically to efficiently degrade chains of cellulose. Whereas EGs and BGs are quite common in microorganisms, animals and plants, CBHs are apparently more rare and appear to be restricted to bacteria, fungi and protists [59,60]. In our dataset, 9 contigs were assigned to putative EGs and 11 contigs were assigned to putative BGs (Fig 6). However, we found no evidence for the presence of genes encoding CBHs.

Using CAZymes Analysis Toolkit [47], 9 families of putative genes of Carbohydrate-Active Enzymes (CAZy) were detected in the 3 transcriptomes (Table 4): 5 glycoside hydrolases (GH1, GH9, GH13, GH37, GH45), 2 glycoside transferases (i.e., GT3, GT35) and 2 carbohydrate-binding modules families (i.e., CBM6, CBM48). Genes of a same GH family are usually considered to share not only structural motifs and the catalytic machinery, but also an evolutionary origin [61]. Among the putative GH genes detected in our analyses, GH1 represents a single family of BGs, whereas GH9 and GH45 are two families of EGs. Previous studies show GH1 and GH9 to be mostly expressed by the genome of termites either in their salivary glands and/or in the hindgut [62]. However, genes encoding GH45 can be expressed by both the hosts and their symbiotic protists or prokaryotes living in the hindgut. In addition, CBMs are usually considered to be expressed by microorganisms only (Watanabe & Tokuda 2010). Therefore, the detection of putative genes encoding GH45 and CBM in our dataset suggests that the gut microbial community of neotenics may play a role in the synthesis of the enzymatic system involved in the degradation of cellulose.

**Table 4:** Number of Carbohydrate-Active Enzyme (CAZy) families detected in *Reticulitermes* transcriptomes.

CAZy Families	Pfam domains	Domain descriptions	Putative enzymatic activities	*R. flavipes*	*R. grassei*	*R. lucifugus*
CBM48 /GH13	CBM_48/Alpha-amylase/Alpha-amylase_C	Carbohydrate-binding module 48 (Isoamylase N-terminal domain)	-	1	0	1
CBM6	Phosphodiest	Type I phosphodiesterase / nucleotide pyrophosphatase	-	1	1	0
GH1	Glyco_hydro_1	Glycosyl hydrolase family 1	3.2.1.21	4	0	1
GH13	Alpha-amylase	Alpha amylase, catalytic domain	3.4.1.183.2.1.1	1	0	0
GH37	Trehalase	Trehalase	3.2.1.28	2	2	3
GH45	Glyco_hydro_45	Glycosyl hydrolase family 45	3.2.1.4	1	1	0
GH9	Glyco_hydro_9	Glycosyl hydrolase family 9	3.2.1.4.	1	1	2
GT3	Glycogen_syn	Glycogen synthase	2.4.1.11	2	1	1
GT35	Phosphorylase	Carbohydrate phosphorylase	2.4.1.1	11	3	3

### Interspecific variation of microbial gene expression patterns

The proportion of microbial contigs varies among the three *Reticulitermes* species (Table 3). This variation is particularly evident in the contigs assigned to protists. Representing only 0.3% of the sequences in *R*. *lucifugus*, protists contigs were more abundant in *R*. *grassei* (2.1%) and in *R*. *flavipes* (6.4%). This general pattern has been found in the functional analysis also since the most important proportion of microbial contigs was assigned in *R*. *flavipes* transcriptome followed by those of *R*. *grassei* and *R*. *lucifugus* (Figs 2–4). We cannot exclude that a part of this observed variation results from methodological fluctuations in mRNA isolation, cDNA library construction and sequencing. However, this pattern might also result from variation of gene expression patterns among individual neotenics of different species, either due to transcriptomic noise or associated to a biological function. In any case, our results suggest that gut microbial communities are not totally absent from *Reticulitermes* neotenics, in spite of their feeding lifestyle, which probably does not directly involve lignocellulose digestion. Abundance, role and regulation mechanisms of gut microbial communities in reproductive termites will require further investigations.

## Conclusion

Comparison of a set of assembled transcriptomes of nymphoid neotenic reproductives was performed from 13 colonies belonging to 3 related termite species based on high throughput Illumina sequencing. Intraspecific variation was addressed by pooling two to nine individuals per species. As expected with non-model organisms, a large fraction of contigs had no detectable homologs in the public database. The majority of recovered transcripts had a termite origin, although transcripts from microorganisms provided evidence for the presence of an active gut microbiome in this non-wood feeding life stage. These transcripts were indeed over-represented in starch and sucrose metabolism pathways, and some of them are likely to encode enzymes involved in cellulose degradation.

## Supporting Information

S1 Table Contigs putatively involved in enzymatic activities linked to the starch and sucrose metabolism pathway and detected by GO terms analysis. (DOCX)

S1 Dataset Amino acid sequence of contigs putatively involved in enzymatic activities linked to the starch and sucrose metabolism pathway and detected by GO terms analysis in FASTA format. (DOCX)
