# Supplementary material for: Comparative Analysis of Transcriptomes from Secondary Reproductives of Three Reticulitermes Termite Species
Source: PLoS One. 2015 Dec 23;10(12):e0145596. doi: 10.1371/journal.pone.0145596 (PMC4689415; doi:10.1371/journal.pone.0145596)
Supplement: S1 Table — (DOCX) [file pone.0145596.s001.docx]

**S1 Table:** Contigs putatively involved in enzymatic activities linked to the starch and sucrose metabolism pathway and detected by GO terms analysis.

| **Termite species** | **Enzyme ID** | **Enzyme name** | **No of unigenes of enzyme** | **Predicted proteins of unigenes** |
| --- | --- | --- | --- | --- |
| R. flavipes | ec:2.4.1.1 | phosphorylase | 14 | Contig3835_1, Contig4371_1, Contig10548_1, Contig34736_1, Contig48119_1, Contig78085_1, Contig102352_1, Contig139502_1, Contig164320_1, Contig212758_1, Contig218172_1, Contig302727_1, Contig332820_1, Contig349337_1 |
|  | ec:2.4.1.11 | glycogen(starch) synthase | 2 | Contig50822_1, Contig263043_1 |
|  | ec:2.4.1.18 | 1,4-alpha-glucan branching enzyme | 2 | Contig101609_1, Contig119817_1 |
|  | ec:2.7.1.1 | hexokinase | 4 | Contig68799_1, Contig267937_1, Contig332230_1, Contig332231_1 |
|  | ec:3.2.1.1 | alpha-amylase | 1 | Contig216932_1 |
|  | ec:3.2.1.21 | beta-glucosidase | 7 | Contig64397_1, Contig283554_1, Contig289734_1, Contig322184_1, Contig333730_1, Contig368545_1, Contig374743_1 |
|  | ec:3.2.1.28 | alpha,alpha-trehalase | 2 | Contig277043_1, Contig304485_1 |
|  | ec:3.2.1.33 | amylo-alpha-1,6-glucosidase | 1 | Contig147456_1 |
|  | ec:3.2.1.4 | cellulase | 4 | Contig7394_1, Contig117744_2, Contig218952_1, Contig352330_1 |
|  | ec:3.6.1.9 | nucleotide diphosphatase | 1 | Contig91310_1 |
|  | ec:5.3.1.9 | glucose-6-phosphate isomerase | 3 | Contig22618_1, Contig211082_1, Contig306521_1 |
| R. grassei | ec:2.4.1.1 | phosphorylase | 4 | Contig205800_1, Contig510511_1, Contig518755_1, Contig524123_1 |
|  | ec:2.4.1.11 | glycogen(starch) synthase | 1 | Contig522161_1 |
|  | ec:2.7.1.1 | hexokinase | 4 | Contig172544_1, Contig227517_1, Contig437682_1, Contig494784_1 |
|  | ec:3.2.1.21 | beta-glucosidase | 1 | Contig261629_1 |
|  | ec:3.2.1.28 | alpha,alpha-trehalase | 2 | Contig78073_1, Contig419536_1 |
|  | ec:3.2.1.4 | cellulase | 3 | Contig74988_1, Contig280625_1, Contig424988_1 |
|  | ec:3.6.1.9 | nucleotide diphosphatase | 1 | Contig92994_2 |
|  | ec:5.3.1.9 | glucose-6-phosphate isomerase | 2 | Contig298067_1, Contig300110_1 |
| R. lucifugus | ec:2.4.1.1 | phosphorylase | 4 | Contig185587_1, Contig261215_1, Contig278689_1, Contig373089_1 |
|  | ec:2.4.1.11 | glycogen(starch) synthase | 1 | Contig455311_1 |
|  | ec:2.4.1.18 | 1,4-alpha-glucan branching enzyme | 1 | Contig121835_1 |
|  | ec:2.7.1.1 | hexokinase | 2 | Contig86821_1, Contig235336_1 |
|  | ec:3.2.1.21 | beta-glucosidase | 3 | Contig10951_1, Contig415346_1, Contig431315_1 |
|  | ec:3.2.1.28 | alpha,alpha-trehalase | 4 | Contig6289_1, Contig100926_1, Contig118930_1, Contig399136_1 |
|  | ec:3.2.1.33 | amylo-alpha-1,6-glucosidase | 1 | Contig104036_1 |
|  | ec:3.2.1.4 | cellulase | 2 | Contig168347_1, Contig446659_1 |
|  | ec:5.3.1.9 | glucose-6-phosphate isomerase | 2 | Contig47295_1, Contig165115_1 |
