# Supplementary material for: Comparative Analysis of Transcriptomes from Secondary Reproductives of Three Reticulitermes Termite Species
Source: PLoS One. 2015 Dec 23;10(12):e0145596. doi: 10.1371/journal.pone.0145596 (PMC4689415; doi:10.1371/journal.pone.0145596)
Supplement: S1 Dataset — (DOCX) [file pone.0145596.s002.docx]

**S1 Dataset:** Amino acid sequence of contigs putatively involved in enzymatic activities linked to the starch and sucrose metabolism pathway and detected by GO terms analysis in FASTA format

*Reticulitermes flavipes*

>Contig3835_1

FEGKELRLKQEYFMCAATLQDITRRFKSSKFGCRNPVRTSFDTFPEKV

>Contig4371_1

NILSVIDRYLRILNNPKDKSEFVPRVIIFGGKAAPGYYAAKKLIKLINNVSYVINADPDVGDLLK

>Contig7394_1

AAVISGWGESQACCSCMELTFTSGPISGKKLLVQVTNTGGD

>Contig10548_1

FIDSLATLNLPGWGYGLMYSFGMFKQTIGPDGSQMEIPDYWLDF

>Contig22618_1

FAGSDFAQFIKGMGYMDTLTRRETNNPAAIIAALIYALKEKIGFKNMIVLCYSEFMREFAHYLQQLYMESLGKEYKIDGSPAHVGQTVFGGVGTGEQHSFMQQVQKGLSDCYVRLIFFEKRHEDFVNQQAGSMGRQH

>Contig34736_1

DFYELSPEKFQNKTNGITPRRWLLLCNPSLADLIAEKIGEDWIVHLEQLQQLTQFAKDANFQRAVQKVKQENKLKLAQLLQKEYGVKVNPASMFDIQVK

>Contig48119_1

RKPTVSYPVFFYGRSENGIWHPSLTIIAVANDFLIP

>Contig50822_1

HEFVRGHFYGHYDFDLDKTLYFFIAGRYEFGNKGADVFIEALARLNHYLKSSHSDTTVVAFLIFPAVTNNFNVESLRGHAVTKSLRDTLNDIQQNMGKRMYEVCLSGQLPNPNELLQKDDMVKIKRCIYALQRDGLPPITTHNVVDDWKDPVLNAVRRCQLFNTIHDRVKIVFHPEFLSSTNPLFGLDYEEFVRGCHLGVFPSYYEPWGYTPAECTVMGIPSITTNLSGFGCFMQEHIADPMSYGIYVVDRRYIALEASIQQLAQYMYDFTKLNRRQRIIQRNRTERLSDLLDWRNLGIYYRQARMRALQKVYSDLDLDDDASVGRFNYPRPISEPSSPSSSRHTTPAASRHGSDDEDEVDEEKELEELGVNSNDHASR

>Contig64397_1

PAFVDTGYADIQNEKYPTASSCWLQVVPWGFRKMLNWAAKEYKNPPVLVAENGFSDHGEV

>Contig68799_1

VGTGSNACYVEKVENAELFDGDRSKPYVVINTEWGAFGDDGRLDIVRTEYDREIDQYSINPGKQLHEKMISGMYMGELARLIIVKFTKQGLLFGGKGSDLLFKRGNFFTKYVSEIESDKRGSYVNCRQVLDELGLSHATDQDCSNVRYICECVSKRAAHLVSAGVATLINKMGKDNVTVGIDGSVYRFHPHFHDLMSKKIKQLVTPGIEFDLMLSEDGSGRGAALVAAVACRDR

>Contig78085_1

DYYQAVEMKQRCENLTSVLYPNDNTPEGKEMRLMQEYFLSSATLQDIIRKLKTKQKAPIEDLPKYAACQLNDTHPAIMVAELMRLLMDEENLPFDKALNIAREVFSYTCHTLMPEALEKWSVPLFENLLPRHLEIIYEINYHFLTTTQIPKEDIGAVSIVEESNPKQIRMANLAVVGSHKVNGVAFIHTELMKEFVFNAFYKIWPEKFCNKTNGV

>Contig91310_1

KLTDALSRHPLLMIVSFDGFRHDFFMKRATPGLDRFKSDGTHAPYVRNVFPTKTFPNHHSIATG

>Contig101609_1

CHTYAEFERDILPMVVKLGYNTVQIMAIMEHAYYASFGYQITNFFAVSSRFGTPEELKSLIDKAHSLGLRVFLDLIHSHASKNVDDGINQFDGTDHQYFHGGGRGYHDLWDSRLFNYNHIEVQRFLLSNLKMFLEE

>Contig102352_1

NSELDEEKFRFGDYYQAVEMKQRCENLTSVLYPNDNTPEGKEMRLMQEYFLSSATLQDILKTLKTKQHAPIEDLPKYAACQLND

>Contig117744_2

MRDCLVLSMVATLAAVVAASDYNYSHVLELSLLFYEAQRSGKLPIDNRIPWRGDSALADRGQNGEDLTGGYYDAGDFVKFGFTMASTTTLLSWGYLSYRDAYEAAGQAKYALSAIKWAGDYFIKCHVSPNELYGQVGDFNLDHTFWGRPEDLNMSRPAYKIDTQHPGSDLAGETAAALAAVSLVFRDVNPGYSSKCLEHAKQLYRFASQYRGLYHEAIKGAAQYYESTDYGDELTWAAAWLYKATDDPQFLDEAEHHYMKFRLKERPNEFFYNKKVAGVQILLAQLTRQPDYMEAAQAFC

DFTVSYQKKTPKGLVYIDKFGTLCHAANVAFVCLQAADAGISPSKYRDFARQQIDYMLGDSGRSYVVGFGHNYPKQPHHAASSCLMRPAPCGWDAFNWPRENPQLLKGALVSGPDENDHYMDKREEYVYNEVTLDYNAGFQSAVAGLRHLQLEAFENNRT

>Contig119817_1

GRSGLPRVEVSGEGEKTFKMGGKLSALDPMQVEVPNLNVLLDRDSYLRPYEREFRRRYACFQDYVNKVKEWEGGLDNFTQGYKYFGIHVNSDNSVTAREWAPGAVQVYLTGDFNGWDREGHPYKKLEYGKWEIQIPANADGSCPIAHLSEVKVAIRTKTGELVDRLSPWAVYVVQPPRAEGFTYKQRFWNPPSHEKYQFKHDRVKKPLSLRIYECHVGIATEEPRVGTYKEFGTNIIPRIVKQGYNAIQLMAIMEHAYYASFGYQVTSFYAASSRYGTPEELKELIDTAHAHGLYVMLDVVHSHASKNVLDGLNQFDGTNSCYFHDGARGEHSLWDSRLFNYSEYEVLRFLLSNLRWYAEEYRFDGFRFDGVTSMLYHSRGIGEGFSGHYDEYFGLNVDTEALVYLMLANHMLHELYPDTVTIAEDVSGMPATCRPVSEGGLGFDYRLGMAIPDQWIKLLKEVKDEDWNIGNIVHTLTNRRWQEKTVSYAESHDQALVGDKTIAFWLMDKEMYTHMSTVSEPSGIIDRGLALHKMIRLITHGLGGEAYLNFMGNEFGHPEWLDFPRAGNNDSYHYARRQWHLVDDEILKYKFLNAFDANMNSLEERHGWLHKDSGYVSWKHETDKVMAFERAELLFVFNFHPTQSFTDYRVGVEVPGEYKVALDTDDKKYGGFHRVDSNVPHFTFPEDFAGRKNSIKLYLPSRTAQVYARV

>Contig139502_1

YIKCQDRVSAVYQDQSKWVEMAIHNIASSGKFSSDRTITEYAREIWGVEPTWEKLPAPHEPREEEPAAK

>Contig147456_1

NYGNSKEDNPWLWDYMGQYIRSVSETAHALRIDNAHSTPLHVAEYFIKEARKVNPSMYVMAELFTGGED

>Contig164320_1

DLLKVVFIPNYNVSNAEIIIPGTDVSQHISTAGYEASGTSNMKFAFNAGLIIGTHDGANIEIG

>Contig211082_1

WQKLQQYYNDNGSKINIYQLMKENPNRFDNFSFALSTPQDGDILIDYSKNRINDEVFSLLVDLAKARKVEAARNSMFSAEKINFTENRAVLHVALRNCSNKPIVLDGRDVMPDVNAVLAHIKDFTEEVLSKKWRGYTGKAIEDVVNIGIGGSDLGPLMVTEALKPYTTGLKVHFVSNIDGTHLAETLKKLNPETVLFIIASKTFTTQETITNATSAKHWFLETAKDPSAVAKHFVALSTNGPKVKEFGIDEQNMFGFWDWVGGRYSLWSAIGLSISLYVGFDNFKKLLEGAHYMDNHFQNAPLEKNAPVILALLGIWYSNFYGAETHALLPYDQYLHRFAAYFQQGDMESNGKYVTRSADRVTYNTGPIVWGEPGTNGQHAFYQLIHQGTRLIPADFIAPAQTHNPIQAGLHHKILLANFLAQTEALMKGKTKEEARSELEKSGLSGASLEKILPHKVFEGNRPTNSIVVRKITPFTLGALIALYEHKIFTQGVIWDINSYDQWGVELGKQLAKAIEPELSDKNPVTSHDSSTNGLINFIKKSASSG

>Contig212758_1

SFGMFKQTIGPDGSQMEIPDYWLDFGDPWRIRKPTVSFPVYFYGHTENGVWHPSLTIIAVANDFLIPGFATDNTLALRLWSSRPNSELDEEKFRYGDYYQ

>Contig216932_1

GSRPFIYQEVIDLGGEAVSSSEYTGFGRVTEFKYGAKLGNAFRGNDPIKYLQNFGEPW

>Contig218172_1

FHQKLKDAPEQEHETAKHKLLWKLMGSYVPANKDAVQGSFVSHLEYTVARSRFNFDSFSSYLSASYSVRDRLIELFNDTQEY

>Contig218952_1

ANLSNIQVKKIKGAVSGSGKTTRYWDCCKPSCSWTGKATVSSPVKSCGKDGSASVQDEKSGCDGGTSYMCADQIPRAVNDSYAIGFAAAVISGWGE

>Contig263043_1

MSRDRASRRFYRVESQNELLAYMDKGEMASCQNRWTFEAAWEVANKVGGIYTVIRSKAYVSTEEMGDQYCLLGPYKEHSARTEVEESDFTGPLHIAVSKMREQGFKIHTGMWLVDGNPQIILFDIGSGAWKMDVYKQELWNKCNLGIPHLDVEANDAVILGYMVAQFIEEFRKVAEEYSDYVPRIVAHFHEWQAGVGLIALRTRHVDVATVFTTHATLLGRYLCAGNIDFYNNLDKFAVDEEAGKRQIYHRYCMERAASHLAHVFTTVSEITGYEAEHLLKRKADVITPNGLNVKKFSALHEFQNLHAISKDKIHEFVRGHFY

>Contig267937_1

IREHCKELQLSDVQISEMMNRLLEDIKLGLGKDTHEKSIVKCFVTYVQDLPNGTERGKFLALDLGGTNFRVLLIMLGENKHFDMKSKIYAIPQSIMLGSGAQLFDHIAECLANFMKEQNLGEERLPLGFTFSFPLMQKGLTKGLLERWTKGFDCSGCVGEDVVQLLKDALSRRND

>Contig277043_1

NPSPADISKFVDETFDPPGSEFEDWDPIDWVPYPKFLNNIRDLVLREWARHLHLFWKELGRQMKHEVRNNTELYS

>Contig283554_1

NDTDRVDFYVKYISELLKAINEDGCDVLGYSAWSLMDNFEWVSGYTETFGLFHVDFN

>Contig289734_1

FKAVVDAGVSSVMVNSGLINGIPVHSSKFLLTDILKGELGFEG

>Contig302727_1

SFDTFPEKVAIQLNDTHPSLAIPELMRILVDVEGLTWEKAWDITVRSCAYTNHTVLPEALERWPTSMLHSILPRHLQIIYDINHLHLQEVQKKFPGDWERMKRMSLVEEEGEKRVNM

>Contig304485_1

IIYVQHPVIVPGGRFREFYYWDSYWIIRGLLLSEMHKTVKGMLENFLSMVNKYGFIPNGGRIYYTRRSQPPLLIPMVKSYVDATNDIEFLRKNIDTIEEEFQYWMKNHSVVVVKDGKGYTLARYSAPSSGPRPESYREDYENAQVFRTEAERQNHYTQLKTAAESGWDFSTRWYITNSTHKGSLTDTMTQYIIPVELNAILCWNAQLLSEFYNTLDMANKTVQYRRLADLWLEAVDNVLWHDEVGIWLDYDLINGVKHNHFYPTNLAPLWTGCFTRRDLQVGKIMKYLEHSQIMMYLGGIPTSLEHSGEQWDYPNAWPPLQYIMIMALEATEDVWAHDLAVEVATRWVRSNFKTFNESHVMYEKYDATFPGGHGTGGEYVNQIGFGWTNGVILELLDKYSQLLTVEDDLFTTERGWQHRPLCTHSASSSLVAH

>Contig306521_1

RMESGEVVNHTAVEAESEDRQVDHYNHRLAEEKVKGKSLGLTLKIWEDTLQFANKIKSGEIKTSAGKKYENVIFNGIGGSYLGPLMLIIAKYGLDFNTTAKLPVKIYFISNTDSDMFHEITTSINIETSIMVHLSKSGSTSETAGNMQTWAQLVEKKGLVVGEHNAAVTIKDSQLDQTGQKLKFIKIWHMET

>Contig322184_1

MQCECDYFVLLICLSVAVIHAEMSSGDSYKFPEFFMLGSATASYQIEGGWNADGKGVNIWDT

>Contig332230_1

KDALSRRNDTHKIRVAGILNDAVGTLMSCAYKRDSCRIGVVVGTGSNACY

>Contig332231_1

KDALSRRNDVQIDVCAILNDTTGTLMSCAWKKFNCRIGLIVGTGSNACY

>Contig332820_1

QGKKWVNTQVVFAMPYDSPVPGYGNNVVNTLRLWSAKSPVEFNLKFFNDGDYIQAVIDRNIAENISRVLYPNDNFFEGKELRLK

>Contig333730_1

ELIANGIQPMITMYHFDLPQPLQNLGGWTNPVIANYFEDYARVLYANFGDR

>Contig349337_1

KVKQVYYVSIEFLMGRFLRNALLNLEIEDVYKEGLSELDVDLDSLYTEEYDAGLGNGGLGRLAACFIDSLATLN

>Contig352330_1

VQVTNTGGDLGSNQFDLAIPGGGVGIYNGCTSEYGAPSDGWGSRYGGV

>Contig368545_1

AYRLYEREFKDKQGGKVSIVAVCRWIESMTDSKEEEKAAERARQMHIGWVLHPIYS

>Contig374743_1

DNKVNQAGIDYYNKLIDELLANGIEPMITMYHWDLPQAFQDLGGWPNFVLAQYFEDYARILFTNFGDRVKLWLTFNEPLTFMSGYSSDQGFAPAINLTGIGDYLTARTVIYAHSRVYHLYDQLFRMKQRGKVGIALNINWCEPATNSTEDAAACERYQQFKLGIYAHPIFSAEGDYPTVVKERIARNSAAQGYTKSRLPSFTPKQVEYIRGTADFLGLNFYTAYYGKPGEDGDSPSMARDTGIITTQDPGWASSASSWLTVVPWGFRKE

*Reticulitermes grassei*

>Contig74988_1

LQAILLVLGDNGRTTRYWDCCKGSCGWEKKASVTQPIDTCAKDGTTRVASNDTVKSGCDGGTGFMCYDQTPWAVNDNLAYGFAAAAISGGESAACCACFNLTFTSGPVNGKNMVVQVTNTGGDLGSNQFDLAIPGGGVGIYNGCTAQSGAPQDGWGSRYGGVSSRSECSQLPSGLQAGCQWRFDWFQNADNPSISFNQVSCPGELTSKTNCKRL

>Contig78073_1

MTCSLLLVLMCVKCLPPPCESDIYCYGQLLHMVQMSNIFPDSKTFVDMKMKYPGNEIWQRFGELMNKTDNNPSPADISKFVDETFDPPGSEFEDWDPIDWVPYPKFLNNIRDPVLREWARHLHLFWKQLGRQMKHEVRNNTELYSIIYVQHPVIVPGGRFREFYYWDSYWIIRGLLLSEMHKTVKGMLENFLSMVNKYGFIPNGGRIYYTRRSQPPLLIPMVKSYVDATNDIEFLRKNIDTIEEEFQYWMKNHSVVVVKDGKGYTLARYSAPSSGPRPESYREDYENAQV

>Contig92994_2

MARWEFSIESILYIGLLYFNYVGSISDHPVILVVSYDGFRYNYFDKNITPNMNKVRISGSHAEYMRNVFITKTFTNHHSIATGVYPEVHGILGNEVYDPLYRKILHYSDEFWHFSDAVVPIWTLNQKAGEG

>Contig172544_1

GTGSNACYVEKVENAELFDGDRSKPYVVINTEWGAFGDDGRLDIVRTEYDREIDQYSINP

>Contig205800_1

NTLRLWSAKSPVEFNLKFFNDGDYIQAVIDRNIAENISRVLYPNDNFFEGKELRLKQEYFMCAATLQDITRRFKSSKFCRDPVRTSFDTFPEKVAIQLNDTHPSLAIPELMRILVDVEGLTWEKAWDITVRSCAYTNHTVLPEALERWPTSMLHSILPRHLQIIYDINHLHLQEV

>Contig227517_1

IREHCKELQLSDVQISEMMNRLLEDIKLGLGKDTHEKSIVKCFVTYVQDLPNGTERGKFLALDLGGTNFRVLLIMLGENKHFDMKSKIYAIPQSIMLGSGAQLFDHIAECLANFMKEQNLGEERLPLGFTFSFPLMQKGLTKGLLERWTKGFDCSGCVGEDVVQLLKDALSRRND

>Contig261629_1

KDKQGGKVSIVAVCRWIESMTDSKEEEKAAERARQMHIGWVLHPIYS

>Contig280625_1

PTDGIIDWYPGDNEVDIIGMDIYPDRGDHSSQRSYFELCRDVF

>Contig298067_1

WDINSYDQWGVELGKQLAKAIEPELSDKNPVTSHDSSTNGLINFIKKSASSG

>Contig300110_1

QQGDMESNGKYVTRSADRVTYNTGPIVWGEPGTNGQHAFYQLIHQGTRLIPADFIAPAQTHNPIQAGLHHKILLANFLAQTEALMKGKTKEEARSELEKSGLSGASLEKILPHKVFEGNRPTNSIVVRKITPFTLGALI

>Contig419536_1

REDYENAQVFRTEAERQNHYTQLKTAAESGWDFSTRWYITNSTHKGSLTDTMTQYIIPVELNAILCWNAQLLSEFYNTLDMANKTVQYRRLADLWLEAVDNVLWHDEVGIWLDYDLINGVKHNHFYPTNLAPLWTGCFTRRDLQVGKIMKYLEHSQIMMYLGGIPTSLEHSGEQWDYPNAWPPLQYIMIMALEATEDVWAHDLAVEVATRWVRSNFKTFNESHVMYEKYDATFPGGHGTGGEYVNQIGFGWTNGVILELLDKYSQLLTVEDDLFTTERGWQHRPLCTHSASSS

>Contig424988_1

DYFLKAHTAPTEFYGQVGEGDADHAYWGRPEDMTMARPAYKIDAAHPGSDLAGETAAALAAASIVFKDVVSNYSTNLLTHAKQLFDF

>Contig437682_1

EIDQYSINPGKQLHEKMISGMYMGELARLIIVKFTKQGLLFGGKGSDLLFKRGNFFTKYVSEIESDKRGSYANCRQVLDELGLSHATDQDCSNVRYICECVSKRAAHLV

>Contig494784_1

KDALSRRNDTHKIRVAGILNDAVGTLMSCAYKRDSCRIGVVVGTGSNACY

>Contig510511_1

MSLVEEEGEKRVNMAHLCIVGSHAVNGVARIHSDIIKSDIFRDFYELSPEKFQNKTNGITPRRWLLLCNPSLADL

>Contig518755_1

TNIKKSFNRHLHYTLVKDRNVSTPRDYYFALAHSVKDNLVSRWIRTQQYYYEKDPKRVYYLSLEYYMGRSL

>Contig522161_1

HEFVRGHFYGHYDFDLDKTLYFFIAGRYEFGNKGADVFIEALARLNHYLKSSHSDTTVVAFLIFPAVTNNFNVESLRGHAVTKSLRDTLNDIQQKMGKRMYEVCLSGRLPNPNELLQKDDMVKIKRCIYALQRDGLPPITTHNVVDDWKDPVLNAVRRCQLFNTIHDRVKIVFHPEFLSSTNPLFGLDYEEFVRGCHLGVFPSYYEPWGYTPAECTVMGIPSITTNLSGFGCFMQEHIADPMSYGIYVVDRRYIALEASIQQLAQYMYDFTKLNRRQRIIQRNRTERLSDLLDWRNLGIYYRQARMRALQKVYSDLDLDDDASVGRFNYPRPISEPSSPSSSRHTTPAASRHGSDDEDEVDEEKELEELGVNSNDHASR

>Contig524123_1

QFAKDANFQRAVQKVKQENKLKLAQLLQKEYGVKVNPASMFDVQVKRIHEYKRQLLNCLHIIVLYNRIKHNPAASFTPRTVMIGGKAAPGYHMAKKIIKLICSVANVVNNDPIVGDKLKVIFLENYRVTLAEKIMPAADLSQQISTAGTEASGTGNMKFMLNGALTIGTLDGANVEMAEEMGQENIFIFGMTVDQVETLKHQGYNAYDYYNSHPDIKQCIDQIQNGYFSPSNPDEFRDVVDVLLKYDRFFLLADFESYIKCQDRVSAVYQ

*Reticulitermes lucifugus*

>Contig6289_1

DNNPSPADISKFVDETFDPPGSEFEDWDPIDWVPYPKFLNNIRDPVLREWARHLHLFWKELGRQMKHEVRNNTELYSIIYVQHPVIVPGGRFREFYYWDSYWIIRGLLLSEMHKTVKGMLENFLSMVNKYGFIPNGGRIYYTRRSQPPLLIPMVKSYVDATKDIEFLRKNI

>Contig10951_1

MFLSDTGVVNIPNDKYATAASEWLQVVPWGIRKLLNWIAK

>Contig47295_1

APVILALLGIWYSNFYGAETHALLPYDQYLHRFAAYFQQGDMESNG

>Contig86821_1

KDALSRRNDVQIDVCAILNDTTGTLMSCAWKKFNCRIGLIVGTGSNACYVEKVENAELFDGDRSKPYVVINTEWGAFGDDGRLDIVRTEYDREIDQYSINPGKQLHEKMISGMYMGELARLIIVKFTKQGLLFGGKGSDLLFKRGNFFTKYVSEIESDKRGSYVNCRQVLDELGLSHATDQDCSNVRYICECVSKRAAHLVSAGVATLINKMGKDNVTVGIDGSVYRFHPHFHDLMSKKIKQLVTPGIEFDLMLSEDGSGRGAALVAAVACRDR

>Contig100926_1

MTCSLLLVLMCVKCLPPPCESDIYCYGQLLHMVQMSNIFPDSKTFVDMKMKYPGNEIWQRF

>Contig104036_1

APEMFDPVHAWTALKKAEELLLGPLGMKTLDPGDWSYNGDYD

>Contig118930_1

DIEFLRKNIDTIEEEFQYWMKNHSVVVVKDGKGYTLARYSAPSSGPRPESYREDYENAQVFRTEAERQNHYTQLKTAAESGWDFSTRWYITNSTHKGSLTDTMTQYIIPVELNAILCWNAQLLSEFYNTLDMANKTVQYRRLADLWLEAVDNVLWHDEVGIWLDYDLINGVKHNHFYPTNLAPLWTGCFMRRDLQVGKIMKYLEHSQIMMYLGGIPTSLEHSGEQWDYPNAWPPLQYIVIMALEATEDVWAHDLAVEVATRWVRSNFKTFNESHVMYEKYDATFPGGHGTGGEYVNQIGF

GWTNGVILELLDKYSQLLTVEDDLFTTERGWQHRPLCTHSASSSLVAHVISLT

>Contig121835_1

PRVEVSSEGEKTFKMGGKLSALDPMQVEVPNLNVLLDRDSYLRPYEREFRRRYACFQDYVNKVKEWEGGLDNFTQGYKYFGIHVNSDNSVTAREWAPGAVQVYLTGDFNGWDREGHPYKKLEYGKWEIQIPANADGSCPIAHLSEVKVAIRTKTGELVDRLSPWAVYVMQPPRAEGFTYKQRFWNPPSHEKYQFKHGRVKKPLSLRIYECHVGIATEEPRVGTYKEFGTNIIPRIVKQGYNAIQLMAIMEHAYYASFGYQVTSFYAASSRYGTPEELKELIDTAHAHGLYVMLDVVHSHASKNVLDGLNQFDGTNSCYFHDGARGEHSLWDSRLFNYSEYEVLRFLLSNLRWYAEEYRFDGFRFDGVTSMLYHSRGIGEGFSGHYDEYFGLNVDTEALVYLMLANHMLHELYPDTVTIAEDVSGMPATCRPVSEGGLGFDYRLGMAIPDQWIKLLKEVKDEDWNIGNIVHTLTNRRWQEKTVSYAESHDQALVGDKTIAFWLMDKEMYTHMSTVSEPSGIIDRGLALHKMIRLITHGLGGEAYLNFMGNEFGHPEWLDFPRAGNNDSYHYARRQWHLVDDEILKYKFLNAFDANMNSLEDCHGWLHKDSGYVSWKHETDKVMAFERAELLFVFNFHPTQSFTDYRVGVEVPGEYKVALDTDDKKYGGFHRVDSNVPHFTFPEDFAGRKNSIKLYLPSRTAQVYARV

>Contig165115_1

QQGDMESNGKYVTRSADRVTYNTGPIVWGEPGTNGQHAFYQLIHQGTRLIPADFIAPAQTHNPIQAGLHHKILLANFLAQTEALMKGKTKEEARSELEKSGLSGASLEKILPHKVFEGNRPTNSIVVRKITPFTLGALIALYEHKIFTQGVIWDINSYDQWGVELGKQLAKAIEPELSDKNPVTSHDSSTNGLINFIKKSASSG

>Contig168347_1

RSTEPNFHTLTGALVGGPDSNDSYVDSRQDAVQNMVACDFNAAFQSAVATLI

>Contig185587_1

IAQVENVTNIKKSFNRHLHYTLVKDRNVSTPRDYYFALAHSVKDNLVSRWIRTQQYYYEKDPKRVYYLSLEYYMGRSLQNTMINLGIQGACDEAMYQLGLDIEELE

>Contig235336_1

IREHCKELQLSDVQISEMMNRLLEDIQLGLGKDTHEKSIVKCFVTYVQDLPNGTERGKFLALDLGGTNFRVLLIMLGENKHFDMKSKIYAIPQSIMLGSGAQLFDHIAECLANFMKEQNLGEERLPLGFTFSFPLMQKGLTKGLLERWTKGFDCSGCVGEDVVQLLKDALSRRND

>Contig261215_1

MAACFLDSMATLGLAAYGYGIRYEYGIFAQKIKNGEQTEEPDDWLRFGNPWEKARPEYMLPVN

>Contig278689_1

EEEGEKRVNMAHLCIVGSHAVNGVARIHSDIIKSDIFRDFYELSPEKFQNKTNGITPRRWLLLCNPSLADLIAEKIGEDWIVHLEQLQQLTQFAKDANFQRAVQKVKQENKLKLAQLLQKEYGVKVNPASMFDVQVKRIHEYKRQLLNCLHIIVLYNRIKHNPAASFTPRTVMIGGKAAPGYHMAKKIIKLICSVANVVNNDPIVGDKLKVIFLENYRVTLAEKIMPAADLSQQISTAGTEASGTGNMKFMLNGALTIGTLDGANVEMAEEMGQENIFIFGMTVDQVETLKHQGYNAYDYYNSHPDIKQCIDQIQNGYFSPSNPDEFRDVVDVLLKYDRFFLLADFESYIKCQDRVSAVYQ

>Contig373089_1

IDRNIAENISRVLYPNDNFFEGKELRLKQEYFMCAATLQDITRRFKSSKFGCRDPVRTSFDTFPEKVAIQLNDTHPSLAIPELMRILVDVEGLTWEKAWDITVRSCAYTNHTVLPEALERWPTSMLHSILPRHLQIIYDINH

>Contig399136_1

MKEFVERTFVEGNELQAWAPGDWKENPTVLQKIADPDLKEWARDLNLLWKNLSRKMADDVQKNPDLYSIIYVPNGFVVPGGRFREFYYWDTYWIIQGLLLSEMYDTTRGILENFLSMVDKYGFVPNGGRVYYVQRSQPPLLIPMVDSYVTQTHNITFLKNNINLLEEEFHFWVTNRTVTVKKNGNEYKLARYYATSQGPRPESYSEDYNHAEMLPSEKEKEDFYINIKSAAESGWDFSSRWFISNGTDNGYLLDIDTK

>Contig415346_1

DNKVNQAGIDYYNKLIDELLANGIEPMITMFHWDLPQAFQDLGGWPNFVLAQYFEDYARVLFTNFGDRVKLWLTFNEPLTFMSGYSSDQGFAPAINLTGIGDYLTARTVIYAHSRVYHLYDQLFRMKQRGKVGIALNINWCEPATNSTEDAAACERYQQFNLGIYAHPIFSAEGDYPTVVKERIARNSAAQGYTKSRLPSFTPKQVEYIRGTADFLGLNFYTAYYGKPGEYGDSPSMARDTGIITTQDPGWASSASSWLRVV

>Contig431315_1

KDKQGGKVSIVAVCRWIESMTDSKEEEKAAERARQMHIGWVLHPIYS

>Contig446659_1

MRDCLVLSMVATLAAVVAASDYNYSHVLELSLLFYEAQRSGKLPIDNRIPWRGDSALGDRGQNGEDLTGGYYDAGDFVKFGFTMASTTTLLSWGYLSYRDAYEAAGQAKYALSAIKWAGDYFIKCHVSPNELYGQVGDFNLDHTFWGRPEDLNMSRPAYKIDTQHPGSDLAGETAAALAAVSLVFRDVNPGYSSKCLEHAKQLYRFASQYRGLYHEAIKGAAQYYESTDYGDELTWAAAWLYKATDDPQFLDEAEHHYMKFRLKERPNEFFYNKKVAGVQILLAQLTRQPDYMEAAQAFC

DFTVSYQKKTPKGLVYIDKFGTLCHAANVAFVCLQAADAGISPSKYRDFARQQIDYMLGDSGRSYVVGFGHNYPKQPHHAASSCLMRPAPCGWDAFNWPRENPQLLKGALVSGPDENDHYMDKREEYVYNEVTLDYNAGFQSAVAGLRHLQLEAFENNRT

>Contig455311_1

MSRDRASRRFYRVESQNELLAYMDKGEMASCQNRWTFEAAWEVANKVGGIYTVIRSKAYVSTEEMGDQYCLLGPYKEHSARTEVEESDFTGPLHIAVSKMREQGFKIHTGMWLVDGNPQIILFDIGSGAWKMDVYKQELWNKCNLGIPHLDVEANDAVILGYMVAQFIEEFRKVAEEYSDYVPRIVAHFHEWQAGVGLIALRTRHVDVATVFTTHATLLGRYLCAGNIDFYNNLDKFAVDEEAGKRQIYHRYCMERAASHLAHVFTTVSEITGYEAEHLLKRKADVITPNGLNVKKFSALHEFQNLHAISKDKIHEFVRGHFYGHYDFDLDKTLYFFIAGRYEFGNKGADVFIEALARLNHYLKSSHSDTTVVAFLIFPAVTNNFNVESLRGHAVTKSLRDTLNDIQQKMGKRMYEVCLSGRLPNPNELLQKDDMVKIKRCIYALQRDGLPPITTHNVVDDWKDPVLNAVRRCQLFNTIHDRVKIVFHPEFLSSTNPLFGLDYEEFVRGCHLGVFPSYYEPWGYTPAECTVMGIPSITTNLSGFGCFMQEHIADPMSYGIYVVDRRYIALEASIQQLARYMYDFTKLNRRQRIIQRNRTERLSDLLDWRNLGIYYRQARMRALQKVYSDLDLDDDASVGRFNYPRPISEPSSPSSSRHTTPAASRHGSDDEDEVDEEKELEELGVNSNDHASR
